# Incidentally Detected Squamous Cell Carcinoma of Renal Pelvis in Patients with Staghorn Calculi: Case Series with Review of the Literature

**DOI:** 10.5402/2011/620574

**Published:** 2011-04-26

**Authors:** Ayushi Jain, Deepti Mittal, Arpita Jindal, Ranjana Solanki, Suman Khatri, Archana Parikh, Kamlesh Yadav

**Affiliations:** ^1^Department of Pathology, Sawai Man Singh (SMS) Medical College, JLN Marg, Jaipur, Rajasthan 302004, India; ^2^Parikh Pathology Centre, Keshav Nagar, Jaipur, Rajasthan 302019, India

## Abstract

Squamous cell carcinoma of the renal pelvis is a rare neoplasm, often unsuspected clinically due to its rarity and ambiguous clinical and radiological features, and hence patients present at advanced stages resulting in poor prognosis. We report here four cases of incidentally diagnosed primary renal squamous cell carcinoma, treated at our hospital over a short span of one year, and review the relevant literature. Mean age of the patients (3 males, 1 female) was 60 years. All suffered from staghorn stones. Interestingly, renal carcinoma was unsuspected clinically in all patients. In one case, a computerised tomography scan showed a suspicious nodule. All underwent nephrectomy for nonfunctioning kidney. In just two cases, tumor was identified on gross examination, while the other two only showed thickened pelvis. Our series emphasises the need for pelvicalyceal biopsy during treatment for long-standing nephrolithiasis, and thorough sampling of the renal pelvis in nephrectomy specimen of such patients.

## 1. Introduction

Primary renal squamous cell carcinoma (RSCC) is a rare cancer with a variable incidence of about 0.5–15% of all urothelial cancers [1–4]. There are only isolated case reports and scant case series of such cases in the English literature [1–8]. Herein we report four cases of incidentally detected renal pelvis squamous cell carcinomas in patients having a history of staghorn renal calculi with hydronephrosis. Three of the patients were operated for renal stones in our hospital while one was referred to us for confirmation of diagnosis and further management. With the exception of the latter, in whom a suspicious lesion was seen on imaging studies, in all the rest a carcinoma was unsuspected clinically as well as radiologically, and the diagnosis came to light only on histology. In one case, the whole kidney was infiltrated with RSCC; however, there was no suspicion of a tumor on radiology even retrospectively. Our series highlights the need for a renal pelvic biopsy and periodic radiological evaluation in patients undergoing treatment for renal stones, as RSCC usually escape detection, with dire consequences for the patient, and are only identified incidentally at late stages, when the patient undergoes surgery for a nonfunctioning kidney.

## 2. Case Series

**Case 1**

A 50-year-old male patient presented with pain in the right flank, off and on for two months. Examination of the abdomen was unremarkable. Urine examination revealed mild hematuria and 2+ proteinuria. The blood urea was 43 mg/dL and serum creatinine 1.4 mg/dL. The total glomerular filtration rate (GFR) was 80% with 11.2% on the right side and 89% on the left side. X-ray of the kidney, ureter and bladder (KUB) showed presence of bilateral renal calculi with presence of staghorn calculi in the right kidney and multiple small calculi in the left kidney. Ultrasound evaluation showed right renal hydronephrosis with calculi. The patient underwent a right nephrectomy for right-sided nonfunctioning kidney. On gross examination the kidney was enlarged and showed a dilated pelvi-calyceal system, presence of calculi in the lumen, and thinning of the renal parenchyma to a narrow peripheral rim. No corticomedullary distinction was identified. The renal pelvis appeared pale and diffusely thickened. The resected free end of ureter showed presence of necrotic material in the lumen. Extensive sampling of the thickened pelvis was done and microscopic examination showed a well-differentiated squamous cell carcinoma (Figure 3(a)), infiltrating the renal parenchyma and surrounding the perirenal fat (Figure 3(g)). Thus the stage was—Stage III (pT3N0Mx). The patient had an uneventful postoperative course but was lost to follow up after discharge from the hospital before his metastatic workup could be performed.

**Case 2**

An 87-year-old man presented with pain in the left lower abdomen since the last two months, increasing since the past few days. He had a history of percutaneous nephrolithotomy (PCNL) for bilateral renal stones 2 years before. On examination there was a visible lump on the left loin which was ballotable. His urine output was reduced to 600–800 mL/day. The patient had a history of hypertension and coronary artery disease. He had a serum urea level of 77 mg/dL and serum creatinine level of 2.8 mg/dL. Ultrasound examination revealed left nephrolithiasis with staghorn calculi and hydronephrosis. Left-side nephrectomy was undertaken in view of pyonephrosis. Grossly the kidney was markedly enlarged with marked dilatation of the pelvicalyceal system and virtually absent residual renal parenchyma (Figure 2(a)). A single staghorn stone was identified in the dilated calyces. At the upper pole, a pale area of thickening was identified measuring 4 × 2 × 2 cm. Histological examination of the thickened area in pelvis showed a well-differentiated squamous cell carcinoma infiltrating the perinephric fat. Perineural invasion was also seen (Figure 3(f)). Two lymph nodes identified at the renal hilum showed metastasis. With these features, the stage was—Stage IV (pT3pN2cM0). The patient had coronary complications after surgery and died in the hospital.

**Case 3**

**Figure 1:**
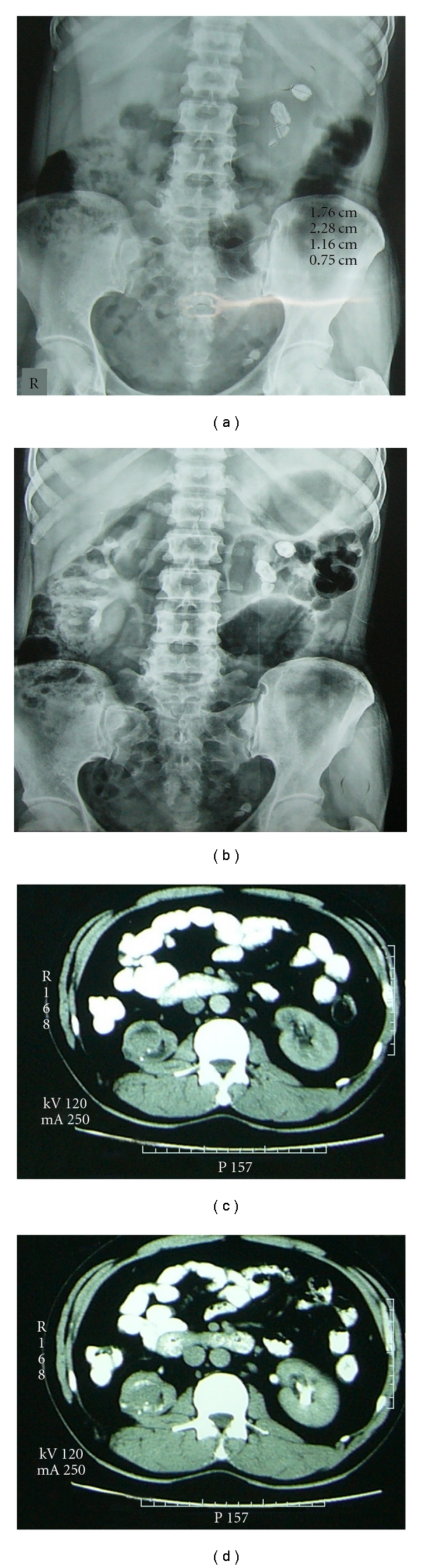
Radiological images. (a) Plain X-ray (KUB) of Case 3 showing multiple radio-opaque shadows in the left kidney; (b) IVP skiagram of the same case at 15 min showing normal excretion of dye in the right kidney while there is complete absence of excretion in the left kidney. Dye excretion was not seen in the left kidney even up to 24 hours. Multiple renal stones can be seen in the left kidney similar to (a); (c) CT scan image of Case 4 showing small and irregular hydronephrotic right kidney with a nodule in the region of pelvis; (d) CECT image of Case 4 showing focal contrast enhancement in the nodule in pelvis.

**Figure 2:**
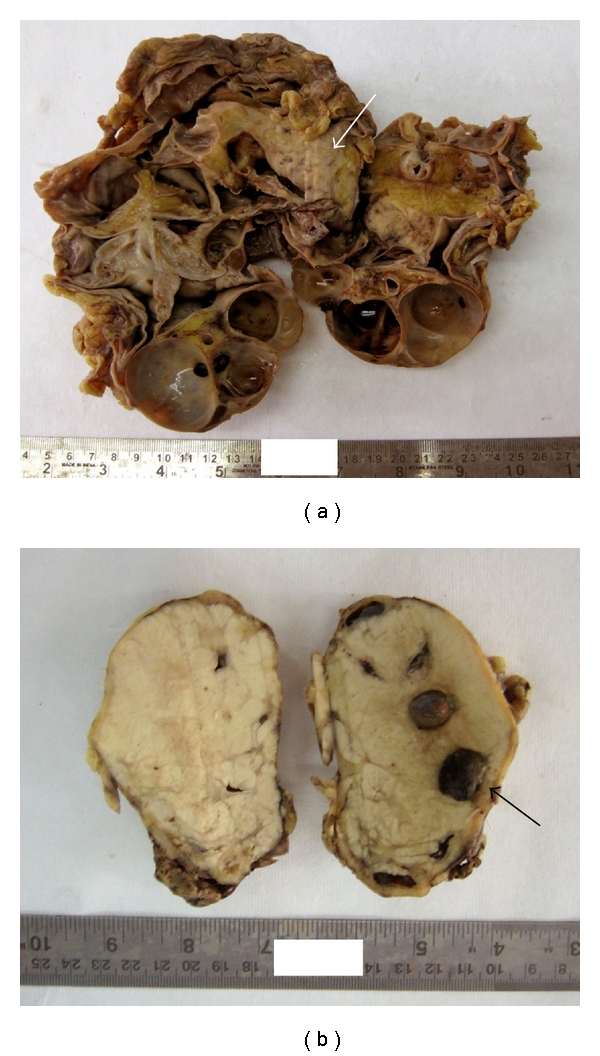
Gross photographs of operated kidney specimen showing (a) marked hydronephrosis, dilated pelvicalyceal system with markedly thinned out renal parenchyma seen in Case 2. An area of thickening is seen at the upper pole (white arrow); (b) replacement of the whole kidney by a solid grey-white tumor in Case 3. The tumor is limited to the kidney. In situ renal stones are identified (black arrow) and there are features of hydronephrosis.

**Figure 3:**
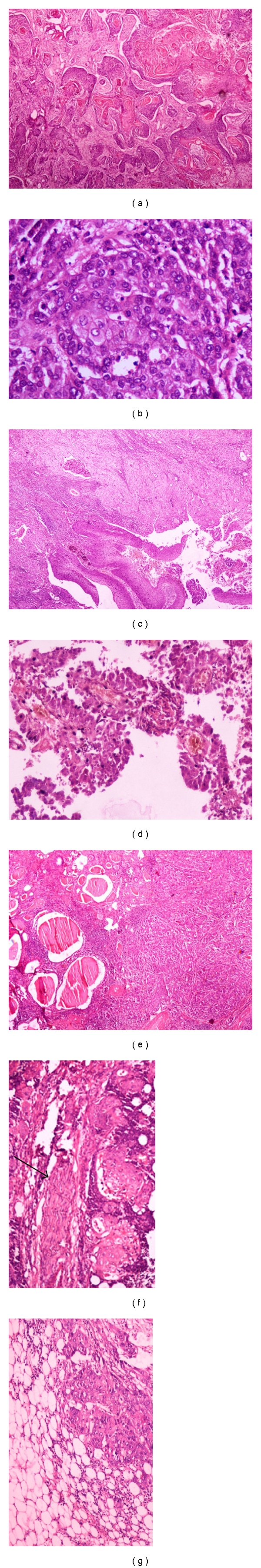
Microphotographs showing (a) well-differentiated SCC with keratin pearl formation seen in Case 1 and (b) poorly differentiated squamous cell carcinoma seen in Case 3. The tumor is comprised predominantly of undifferentiated malignant tumor cells with islands of clearly squamous cells interspersed without keratinisation as seen in this picture; (c) moderately differentiated SCC in Case 4 showing solid sheets of tumor cells arising from the pelvis and infiltrating renal parenchyma. The urothelium lining the pelvis shows squamous metaplasia and dysplasia; (d) the same case showing tumor cells forming true papillae with fibrovascular cores seen in other areas; (e) all cases showed features of chronic pyelonephritis in the surrounding kidney as can be seen here in Case 3; (f) perineural invasion (arrow) of tumor cells seen in Case 2; (g) section from Case 1 with infiltration of the perirenal fat by the tumor cells.

A 50-year-old female patient presented with complaints of pain in the left flank three months ago. Her abdominal examination was unremarkable. There was reduced urine output (600–700 mL/day); however, there was no hematuria. X-ray of KUB showed radiopaque shadows in the left kidney and pelvis (Figure 1(a)). Intravenous pyelography (IVP) revealed non functioning left kidney (Figure 1(b)). Ultrasound evaluation showed left renal and ureteric calculi with absence of corticomedullary distinction. The right kidney was normal. No pre- or paraaortic lymph nodes were visualised. The patient underwent left nephrectomy, and on gross examination, the kidney was normal sized; however, showed few dilated calyces with lodged stones. Surprisingly, nearly the whole kidney was replaced by a solid grey-white tumor, surrounding the dilated calyces, with a scant rim of renal parenchyma seen at the periphery (Figure 2(b)). Microscopy of the tumor revealed a poorly differentiated squamous cell carcinoma (Figures 3(b) and 3(e)). The tumor was confined to the kidney. The patient had an uneventful postoperative course in the hospital and was discharged. The metastatic work-up was negative, and hence she was staged as stage II (pT2N0M0). Currently three months after surgery, the patient is alive and undergoing cisplatin-based chemotherapy.

**Case 4**

A 53-year-old male presented with complaints of bilateral flank pain since the last five months. He was previously operated twice for renal stones, having a right pyelolithotomy 16 years before and a right PCNL seven years before. His biochemical evaluation was normal. Ultrasound KUB revealed right renal calculi with hydronephrosis. Diethylene triamine pentaacetic acid (DTPA) scan showed a small very poorly functioning kidney on the right side. Total GFR was 68.88 mL/min with GFR of the right kidney being 8.09 mL/min and of left being 60.8 mL/min. Contrast enhanced computerized tomography (CECT) scan showed small irregular hydronephrotic right kidney with a suspicious nodular lesion (Figures 1(c) and 1(d)). The left kidney also showed lower calyceal and lower ureteric calculi but with normal renal function. The patient underwent a right nephrectomy at a private hospital. Grossly the kidney was small and showed a dilated pelvicalyceal system filled with pus and fragments of renal calculi. No corticomedullary distinction was seen. A solid grey-white nodular lesion with areas of necrosis, measuring 2 × 1.2 cm, was present near the lower pole. Histopathology of the lesion showed a moderately differentiated squamous cell carcinoma, with solid and focal papillary pattern (Figures 3(c) and 3(d)). The adjacent urothelium showed squamous metaplasia and dysplasia (Figure 3(c)). Infiltration into the perirenal fat and lymphovascular emboli were seen. Tumor emboli were also seen in the renal vein at the hilum. The tumor was hence of stage III (pT3pN0M0). The patient was referred to our hospital for confirmation of the diagnosis and further management. He is alive five months after surgery and undergoing cisplatin-based chemotherapy.

## 3. Discussion

Cancers of the kidney and renal pelvis are the ninth most common malignant cancer and form the 12th most common cause of all cancer-related deaths [9]. Of all urothelial tumors, only 5-6% occur in the upper urinary tract (renal pelvis and ureter), and of these only about 6–15% are squamous cell carcinomas [5]. Among malignant renal tumors, SCC are decidedly rare neoplasms and form only about 0.5–8% [1–3, 9, 10]. Over one year (1st January 2010 to 31st December 2010), 15 patients with hydronephrosis and nonfunctioning kidney were operated at our hospital and one case of such an operation was sent to us for review. Of these 16 cases, 4 showed SCC involving the renal pelvis and kidney (25%). On reviewing all malignant renal tumors diagnosed in our department during the same time period, we found that RSCC comprised 4/21 cases (19%). The remaining cases comprised 11 cases of clear cell adenocarcinoma (52%), two cases of papillary adenocarcinoma (9.5%), and one case each of chromophobe renal cell carcinoma, one case of collecting duct carcinoma, one case of Wilm's tumor, and one case of transitional cell carcinoma (4.8% each). The relatively high incidence of RSCC in our series could be due to the referral pattern of our hospital, which is a tertiary care referral institute.  Table 1 shows the various clinical features and pathological findings of the cases in the present series. 

Most of the current data of RSCC is derived from small case series, over time periods ranging from 6 years to 27 years, and few isolated case reports [1–8]. The predisposing factors leading to development of RSCC are chronic irritation due to preexisting renal stones (most commonly of the staghorn type) or prior surgery for renal stones, analgesic abuse, or radiotherapy. Chronic irritation induced by the aforementioned conditions, superimposed by infection, is believed to induce squamous metaplasia and subsequent development of leukoplakia and neoplasia in the urothelium, resulting in RSCC [1–8, 11]. 

Clinically, the mean age of presentation is 56 years, and contrary to earlier reports, there is equal incidence in males and females. The involvement is unilateral, equally common on the right and left sides. Presenting symptoms include loin pain, hematuria, and abdominal lump [1, 2, 4]. History of previous surgery for renal stone or staghorn calculi in patients of RSCC has been reported variably from only 12.3% of cases in one series to 100% of cases in others [2, 4]. Li and Cheung [2] reported an incidence of RSCC in 2% of patients with recurrent renal stones. In the present series, the mean age was 60 years, M : F ratio was 3 : 1, right side to-left-side ratio was 1 : 1, and most common presenting symptom was flank pain. Hematuria was observed in only one of our patients and may have been due to renal stones. This is similar to the study by Li and Cheung [2]. In all of our cases (100%) there was presence of staghorn type of renal stones. In two of our four cases (50%) there was history of prior surgery for renal stones and three out of four patients (75%) had a history of bilateral renal calculi. 

Significantly, due to nonspecific and insidious presenting symptoms such as flank pain and hematuria, which often overlap with symptoms of renal stones, lack of specific radiological features, and rarity of this tumor, most cases of RSCC are undiagnosed preoperatively and come to light only on histopathological examination of the excised non functioning kidney [2, 3, 5, 6, 8, 12]. A retrospective review of radiological findings in RSCC showed that conventional radiological findings of filling defects, obstructive lesions or nonfunctioning kidney by intravenous urography (IVU), which have been documented sporadically in the literature, are all nonspecific [3]. Because of this, a renal tumor usually remains unsuspected and further radiological evaluation such as computerised tomography (CT) is not done routinely in every case, especially in developing countries, where the cost of these investigations is an issue. Even CT imaging does not help in exact diagnosis, but may provide helpful information regarding the anatomical extent of the tumor [2]. Lee et al. [3] found that the most helpful features in CT of RSCC were presence of enhancing extraluminal and exophytic mass and, in some cases, an intraluminal component. They further suggested, that as it is impractical to perform CT for every patient with renal stone, IVU should be done periodically, especially in long standing stones, and should be read as a split function test for all portions of renal parenchyma. In such cases, filling defects, delay in appearance of pyelogram, or renal parenchymal thickening should be regarded as renal tumor despite the absence of mass effects and preservation of renal contour, warranting further studies by CT or biopsy from renal pelvis or calyces during treatment for renal stones. In the present series, similar to the literature, due to nonspecific clinical and radiological features, there was lack of confirmed preoperative diagnosis of a renal tumor in all our cases. In 3 of our 4 cases, CT was not done preoperatively as clinically, and on conventional radiology such as X-ray, ultrasonography, and IVU, the diagnosis was non functioning kidney with staghorn calculi and hydronephrosis. In 2 of these 3 cases (Cases 1 and 2), gross inspection showed only focal areas of thickening of pelvis which were extensively sampled and microscopic examination proved them to be RSCC. In the third case, even though the diagnosis was evident on gross examination of the resected specimen, as almost the whole kidney was replaced by the tumor, even a retrospective radiological review did not show any evidence of a tumor, much to the surprise of our radiologists. In the fourth case, though no tumor was suspected clinically, a CT scan was performed and revealed a suspicious nodule present intraluminally in the region of the pelvis and histological examination advised. Hence our series underlines the fact that RSCC may be missed, clinically as well as radiologically, in a high number of cases, and the diagnosis may only be incidental during microscopic examination, provided extensive sampling of the kidney, particularly of the renal pelvis, has been done, specially if there is any area of thickening.

Due to the above-mentioned reasons, most patients of RSCC present at late stages, accounting for the poor prognosis [1, 2, 4, 5, 12]. Holmäng et al. [4] compared RSCC with urothelial carcinomas (UCs) and found that though there was no significant difference in prognosis in advanced stage (pT3 and pT4) RSCC and UC, more patients of RSCC presented at advanced stage (94%) than UC (37%). Hence, the outcome of RSCC patients is poor with median survival of only 5–7 months after surgery and less than 10% alive after 5 years [1, 2, 4, 5]. In the present series, similarly three of our four patients (75%) presented at advanced stage (pT3 and pT4). In one of the patients with stage pT3, the nodal status was also advanced (pN2), upstaging the patient to stage IV. Only Case 3, the 50-year-old female, had a lower stage (pT2). 

Histologically, nearly a fourth of RSCC also shows other histological patterns focally including micropapillary, lymphoepithelial, small cell, and sarcomatoid. RSCC with solid and papillary pattern has been seen in 14% of cases in one large series, and most cases are high grade [4]. In our series, one of the four cases (25%) showed a mixed solid and papillary pattern. Two cases were of well-differentiated SCC (50%), one of moderate (25%) and one of poorly differentiated SCC (25%).

Treatment involves surgery with nephrectomy or nephroureterectomy. Radical nephroureterectomy with excision of bladder cuff is the treatment of choice in patients without metastasis; however, in view of the unifocal nature of this disease, parenchyma sparing surgeries have also been proposed [2, 4, 5, 8, 12]. Cisplatinum-based adjuvant chemotherapy and radiotherapy are usually given due to the advanced stage and poor prognosis in most patients but have shown no survival benefit, highlighting the need for early diagnosis [1, 2, 4, 8, 10]. One of our patients was lost to follow up, one died of intercurrent CAD while the remaining two (one pT2 and the other pT3) were on cisplatinum-based chemotherapy and surviving three and five months after surgery, respectively.

## 4. Conclusion

Primary RSCCs are rare tumors and show a strong association with renal stones, which might confound diagnosis. They may not be radiologically detectable and the first indication of malignancy might come incidentally on histological examination of nephrectomy for nonfunctioning hydronephrotic calculous kidney. This emphasises the necessity of prompt treatment of renal stones and assessment for renal tumors in patients with long-standing staghorn calculi. The high incidence of RSCC in hydronephrotic kidneys in our series also highlights the need for meticulous sampling of the renal pelvis by the pathologist in such specimens.

## Figures and Tables

**Table 1:** Patient characteristics of present case series.

Patient characteristics	Case 1	Case 2	Case 3	Case 4
Age (years)	50	87	50	53
Sex	Male	Male	Female	Male
Presentation	Flank pain, mild hematuria	Flank pain, abdominal lump	Flank pain	Flank pain
Duration of symptoms	2 months	2 months	3 months	5 months
Renal stone	Present bilateral	Present bilateral	Present unilateral	Present bilateral
Previous history of stone surgery	Absent	Present	Absent	Present
Hydronephrosis with nonfunctioning kidney	Present	Present	Present	Present
Procedure	Right nephrectomy	Left nephrectomy	Left nephrectomy	Right nephrectomy
Radiologically detected tumor	Absent	Absent	Absent	Suspicion present
Grossly detectable tumor	Absent	Absent	Present	Present
Microscopic grade	Well differentiated	Well differentiated	Poorly differentiated	Moderately differentiated, solid with papillary
Pathological stage	pT3pN0Mx	pT3N2cM0	pT2N0M0	pT3N0M0
Followup	Lost to follow up	Died of CAD	Alive at 3 months*	Alive at 5 months*

*Both patients on chemotherapy.
